# Comparison of levonorgestrel-releasing intrauterine device with oral progestins in heavy menstrual bleeding (HMB) cases with uterine leiomyoma (LNG-IUD and oral progestin usage in myoma uteri)

**Published:** 2014

**Authors:** Ayse Kavasoglu Tosun, Ismet Tosun, Necdet Suer

**Affiliations:** 1 Dr. Ayse Kavasoglu Tosun, Gynecology and Obstetrics, Department Istanbul Medeniyet University, Goztepe Education and Research Hospital, Istanbul, Turkey.; 2 Dr. Ismet Tosun, Gynecology and Obstetrics Department, Umraniye Education and Research Hospital, Istanbul, Turkey.; 3 Associate Professor Dr. Necdet Suer, Gynecology and Obstetrics, Department Istanbul Medeniyet University, Goztepe Education and Research Hospital, Istanbul, Turkey.

**Keywords:** Anemia, Leiomyoma, LNG-IUD, Heavy Menstrual Bleeding (HMB), Oral progesterone

## Abstract

***Objective:*** To compare the effectiveness and acceptability of LNG-IUD with oral progesterone (norethisterone acetate; NETA) in achieving a reduction in volume of the myomas, hemoglobin levels, satisfaction of the women.

***Methods:*** This study includes randomized 30 women treated by LNG-IUD and randomized 30 women treated by oral norethisterone (NETA). All these participants in the study have received medical treatment and had been registered as patients in Istanbul Medeniyet University Göztepe Education and Research Hospital. Leiomyoma volumes and hemoglobin levels have been determined. In the third and sixth months, these measurements have been done again. We examined the adverse effects and the treatment continuity. For the statistical analysis of the findings NCSS [Number Cruncher Statistical System] 2007 & PASS 2008 program; student t, Mann Whitney U, Paired Samples t, Wilcoxon Signed Ranks, Ki-Kare, Fisher's Exact Ki-Kare tests have been used.

***Results:*** After six months treatment, the reduction of bleeding determined by Visual Bleeding Score (VBS) in LNG-IUD group is 80% and in oral norethisteron group is 56%; in both groups leiomyoma volumes and hemoglobin levels were significantly high.

***Conclusion:*** LNG-IUD is a good alternative treatment to the oral progesterone in long term minimizing the hysterectomy for myoma uteri because of the good patient tolerance and easy usage.

## INTRODUCTION

Uterine leiomyomas are the most common pelvic tumor in women.^1^ Aproximately 20-25% of women have fibroids but in the studies which are assessed by histological and sonographical methods, the frequency is declared as 70-80.^2^ A uterine leiomyoma is usually asymptomatic only 20-50% is symptomatic. The symptoms are abnormal uterine bleeding, disparonia, dismenorrhea, pelvic pain/pressure, adverse pregnancy outcomes and infertility.

Typical bleeding problem in leiomyomas, 80 ml and over blood loss in every cycle is heavy menstrual bleeding (HMB) or hypermenorhea.^3^ In medical treatment the choices are NSAID, progesterones, gestrinone, mifepristone, danazol, oral contraceptives and GnRH agonists. The patient with myoma uteri have been treated by hysterectomy up to about 35%.

LNG-IUD, although has been developed for contraception, in short term it has proved to reduce menstruel blood loss.^4^ LNG-IUD contains 52 mg 19-norprogesterel levonorgestrel. Daily 20 µgr levonergestrel is released; it shortens the time of bleeding and blood loss by inhibiting endometrial proliferation. In some clinical studies, it has been claimed that HMB was significantly decreased and the sizes of leiomyoma have got smaller.^4^^, ^^5^

The purpose of this study was to compare the effectiveness of LNG-IUD and NETA for the treatment of myoma uteri with bleeding and also patient satisfaction. LNG-IUD does not affect ovulatuar functions and its adverse effects are few. In leiomyoma cases LNG-IUD can be an alternative method to operational treatment.

## METHODS

This study has been carried out at Istanbul Medeniyet University Goztepe Education and Research Hospital Gynecology & Obstetrics Clinics between the dates January 1^st^ 2010 to March 1st 2011. It was designed as an open-label, randomized, parallel group, therapeutic study. The two medical treatment methods were suggested to the women who refused any kind of surgery. Some of them didn’t want to have an intrauterine device. Random sequence methods were used; randomization was undertaken using computational random-number generators. Consequently, not all patients who attended the hospital during the study period were included in the trial, even if they met the inclusion criteria. Each patient was examined by ultrasound. Thirty women were treated by LNG-IUD and 30 women treated by NETA. All women participated voluntarily and involved in the study based upon their policlinics application number. They gave their written informed consent before entry into the study. The study was granted ethical approval from Istanbul Medeniyet University Göztepe Education and Research Hospital Ethics Committee.

The patients who have pelvic inflamatuar disease, malignancy, tromboembolism, pregnancy, submucosal myom having component inside the cavity over 50% and myomas bigger then 5 cm were excluded from the study. To exclude cervical pathology, PAP smear has been done with pipelle not requiring general anesthesia.

The patients gave information about their bleeding concern and their satisfaction after the treatment.The patients were asked, to write down their menstruel period because it is not possible to measure the menstruel blood in laboratory conditions.

**Fig.1:**
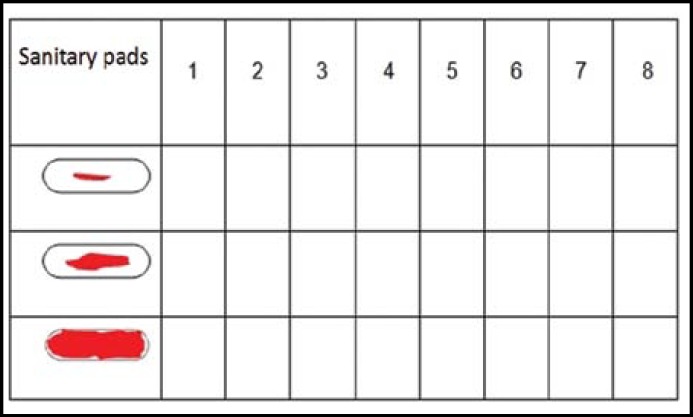
The measurement of menstrual blood loss

To minimize subjectivity, the patients were advised to use the same brand sanitary pads and change them when they get the same amount of dirth. The tecnique developed by Higham and his friends has been used to find out the difference of blood loss between normal menstruel period and HMB.^6^ The evaluation for the menstruel blood loss, the degree of the dirtiness was done by using the form with picture drawings as seen in Fig.1. The numbers 1, 5, 20 have been given for sanitary pads and tampons considering the degree of dirtiness as minimum, middle and heavy. The cut of value in VBS is 185. The predictive value has been found out as 85%.

LNG-IUD has been applied in the first 10 days of the menstruel cycle. The patients have been given oral NETA 10 mg (5mg two times daily) during the cycle of 5-25 days. For the calculation of the volumes of leiomyomas were measured by ultrasonography and the following Formula was used for elipsoid tumor:

Tumor volume=43×π×R1×R2×[R3]

NCSS (Number Cruncher Statistical System) 2007&PASS 2008 program; Student t, Mann Whitney U, Paired Samples t, Ki-Kare and Fisher's Exact Ki-Kare tests have been used. The significance value is p < 0.05. The results are taken from all the patients who continued to participate in the study.

## RESULTS

This study has been carried out between the dates January 1^st^ 2010 to March 1^st^ 2011 on 60 patients; 30 in LNG-IUD group, 30 in NETA group. The age of the patients ranged from 33 to 45, the average age was 39.15 ± 2.79 in LNG-IUD group, parity 2.6 ± 1.1 and in NETA group the average age is 38.91 ± 3.46 and the parity was 2.4 ± 1.1. In LNG-IUD group myom localizations are 9% submukosal, 72% intramural, 19% subserosal; in NETA group 25% submukosal, 60% intramural, 15% subserosal.

In both groups, the level of VBS in the sixth month of study was significantly decreased. The ratio of decrease on the level of VBS in the sixth month of the study in LNG-IUD group was found significantly high to the NETA group (Table-I).

In LNG-IUD group, on the level of hemoglobin in the third and sixth months the significant increase was seen and in NETA group no significant change was seen in the third month. However in the sixth month significant increases was seen. In LNG-IUD group the increase on the hemoglobin level in the third and sixth months have been statistically higher than the levels in NETA group (p < 0.01) (Table-II).

**Fig.2:**
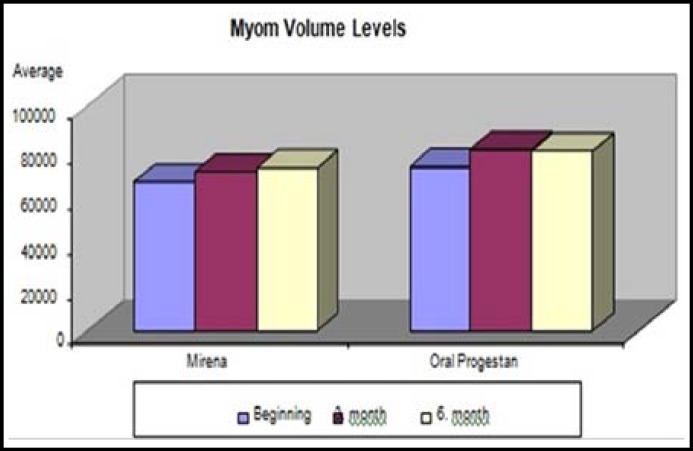
Myom volume levels distribution (mm^3^).

In LNG-IUD group, the myom volume levels have been found significantly high in third and sixth month’s volume compared to the initial level. The sixth month myom volume level has not been found significantly different from the third month’s level. In NETA group, third and sixth month myom volume levels have been significantly high. The levels in the sixth month have not been found significantly different from the third month’s levels (Fig.2).

In both groups menstruation states have not been found statistically different from the initials. When we have done menstruation evaluation in the third month the spotting ratio is high in LNG-IUD group. But in NETA group; normal cycle, HMB, oligomenorrhea and amenorrhea have not been found significantly different. When we did menstruation evaluation in the sixth month, in LNG-IUD group the ratio of amenorrhea was high, in NETA group HMB and normal menstruel cycle ratios was also high. Spotting and oligomenorrhea states have not been found significantly different in both groups (Fig.2).

On each visite the satisfaction of the patients has been questioned. After three months the ratio of satisfaction in LNG-IUD group was 80% and after six months 73%. This ratio for NETA is after three months 50% and at the end of the sixth month 40%.

The attendance of the treatment ratio after three months in LNG-IUD group was 94%, at the end of the sixth month 90%. In NETA group, after three months the ratio is 67%, at the end of the sixth month 60%.

The reasons for dropping out from the treatment in LNG-IUD group were the increase in bleeding and mastalgia in NETA group. Secondly increase in bleeding because of poor compliance with medical therapy. mastalgia, weight gain, depression. The dropping out third month ratio for NETA group was 33.3%, sixth month ratio is 40% and for LNG-IUD third month ratio was 6.6%; sixth month ratio was 10%.

## DISCUSSION

Most of the women with HMB are in the reproductive age, it is important to preserve fertility and their uterus from the physcological aspect for the premenopausal women. Most of the women prefer conservative treatment for this reason.

**Table-I:** Evaluation of VBS (Visuel Blood Scoring).

***VBS***	***Treatment***		***P***
	***LNG-IUD***	***NETA***	
	***Avg±SD***	***Avg ±SD***	
Initial	518,0±120,35	414,33±112,94	0,001**
6. month	77,41±106,15	169,44±166,06	0,028*
^+^Initial-6. month (%) change	80,46±32,66 (87,71)	56,71±40,73 (77,42)	

**Table-II:** Evaluation of Hemoglobin Values

***Hb***	***Treatment***		***p***
	***LNG-IUD***	***NETA***	
	***Avg±SD (Median)***	***Avg±SD (Median)***	
Initial	11,17±1,07	10,69±0,81	0,048*
3. month	11,83±0,98	11,03±0,72	0,004**
6. month	12,73±0,55	11,41±0,75	0,001**
^+^Initial-3. month (%) change	6,90±4,49 (6,19)	2,37±5,89 (2,02)	0,008**
^+^Initial-6. month (%) change	16,09±7,41(17,43)	5,95±7,82 (7,23)	0,001**

**Table-III:** Evaluation of Menstruel Cycle

		***Treatment***		***P***
		***LNG-IUD***	***NETA***	
		***n (%)***	***n (%)***	
Initial Mens	HMB	30 (%100)	30 (%100)	1,000
3-6. months Mens	^●^ HMB	2 (%7,1)	4 (%20)	0,218
	Spotting	18 (%64,3)	6 (%30)	0,0,019*
	^●^Amenorrhea	2 (%7,1)	1 (%5)	1,000
	Normal Cycle	3 (%10,7)	7 (%35)	0,041*
	^●^Oligomenorrhea	3 (%10,7)	2 (%10)	1,000
	HMB Spotting	1 (%3,7) 7 (%25,9)	5 (%27,8) 4 (%22,2)	0,031* 0,777
	Amenorrhea	12 (%44,4)	1 (%5,6)	0,006**
	^●^Normal Cycle	2 (%7,4)	6 (%33,3)	0,045*
	^●^Oligomenorrhea	5 (%18,5)	2 (%11,1)	0,684

In a large number of contrast studies LNG-IUD has proved to provide effective treatment for HMB, endometrial hyperplasia, hormone replasman treatment, endometriosis, adenomyosis and strong contraception effectiveness.^7^

Oral progesterone though used very frequently for the treatment of disfunctional uterine bleeding for over 30 years has proved to have poor effects. Between fifth to 25^th^ days of menstruel cycles 10 mg a day dosage for NETA has decraesed bleeding up to 80-85%. The main problem for progesterone is poor attendance of the patients. In the study carried out by Andrew M and his friends in the sixth month for LNG-IUD group the ratio of attendance to the medical treatment was 77%, for NETA group this ratio goes down to 22%.^8^ In this study the ratio of the patients using NETA was 33.3% at the end of the third month and 40% at the end of the sixth month for the patients dropping out of the treatment. The drop out ratio for LNG-IUD after three months was 6.6% and at the end of the sixth month 10%. In the study group after application, menstruel blood loss for LNG-IUD group was 80%, for NETA group it had reduced by 56%. 

A study done on 32 patients had shown that at the end of the first month of the treatment menstruel blood loss for those using LNG-IUD had reduced up to 80%.^9^ At the beginning, Hemoglobin values were 11,17±1,07 gr/dl in LNG-IUD group, 10,69±0,81 gr/dl in oral NETA group; at the end of the 6 months Hemoglobin values were 12,73±0,55 gr/dl in LNG-IUD group, 11,41±0,75 gr/dl in NETA group. The reduction of HMB gave rise to hemoglobin values. Hemoglobin values increased for LNG-IUD group and it has been found significantly higher than the NETA group. Our findings are parallel to the findings in previous studies^10^. The significant increase of hemoglobin values for LNG-IUD group is closely related to the presence of amenorrhea observed in 44.4%. In another study carried out on 19 patients with myoma uteri, at the end of the one year application hemoglobin value had increased from 11.21 ± 1.80 gr/ dl to 13.44 ± 1.0 gr/dl, similar to our findings^11^.

The most frequent adverse effect of LNG-IUD for the first month is spotting.^12^ The reasons for this are the none-homogen distribution of LNG-IUD at the first months in endometrium and at some point the accurance of splitting.^13^ In our study within the three months eighteen patients (64.3%) had observed this condition. The ratio has dramatically gone down to seven patients (25.9%) at the end of the sixth month. The ratios for NETA group are 30% after three months, 22.2% after six months.

There have been contradictory studies on the effectiveness of LNG-IUD on the volume of uterus and myom. The amount of bleeding has been reported to reduce, but diameter of myom has not reduced.^9^ In another study, the use of LNG-IUD has not always resulted in reduction of the volume of leiomyoma. Furthermore, it sometimes induces potential proliferation on the leiomyoma cells.^13^ Yet, in a different study with the application of LNG-IUD on 67 patients, at the end of the first year the volumes of uterus and leiomyoma got smaller.^14^ In our study we have found out that the release of intrauterine levonorgestrel for the women in the reproductive age has no capacity for the reduction of leiomyoma volume.

The long term effects of LNG-IUD are amenorrhea and oligomenorrhea. In literature the ratios are 20-55%. At the end of the third month of our study in the LNG-IUD group the ratio of amenorrhea was 7.1% (two patients out of 28), at the end of sixth month it was 44.4 (12 patients out of 27); this ratio for NETA group at the end of the third month was 5% (one patient out of 20), after six months 5.6% (one patient out of 18). In both groups no patients stopped taking the medicine. In some studies, patient with leiomyoma and using LNG-IUD the ratio of spotting after three months was 61.2%, oligomenorrhea 16.6%, HMB 5.6%; at the end of the sixth month spotting 24%, oligomenorrhea 36%, amenorrhea 40%, parallel to our findings.^15^

Levonorgestrel has given rise to mastalgia, weight gain, edema rarely hirsutismus, acne, headache.^16^ Because of the adverse effects the number of those dropping out of our study in LNG-IUD group was three patients (10%) after six months. One of them has complained about mastalgia, two reported increase in the amount of bleeding. In NETA group the drop out after six months was twelve patients (40%). One had stopped taking the medicine because of weight gain, one because of mastalgia, eight because of the regular drug usage difficulty and two because of depression.

Excluding the bleeding patterns the most frequent adversive effect was mastalgia for LNG-IUD group, edema for NETA. The reason of mastalgia of levonorgestrel is the release of estradiol from functional persisted over cysts acquiring from partial inhibition of ovulation in the first months. NETA because of the penetrating into the blood circulation it resulted in tense breast. In a study in LNG-IUD group the number of patients complaining from mastalgia at the end of the third month was three (10%), at the end of the sixth month was one (3.3%). One of them had complained about mastalgia, two reported increase in bleeding. Excluding the bleeding patterns for both groups the most frequent adverse effect for LNG-IUD group was mastalgia, for NETA group it was edema. In the other patient at the end of the sixth month the complaints of mastalgia were reduced.

In the studies done by Boon and his friends the patients with the application of LNG-IUD and the patients using NETA at the end of the first year the average weight gain was about 300-500 gram. In this respect there was no difference between LNG-IUD and oral NETA.^17^ In our study both groups frequently complained about weight gain. In both groups at the end of the sixth month the weight gain was 800 grams on average. One patient from NETA group had stopped using the medicine because of this reason. The other frequently seen adverse effect was edema. In our study at the end of the sixth month two patients (6.6%) in LNG-IUD group and six patients (20%) in NETA group had complained about edema.

However, none of the patients had stopped the medical treatment, functional over cysts is mentioned to be one of the most frequently seen adverse effect in literature (10%). In our study in LNG-IUD group at the end of the third month three patients (10%), at the end of the sixth month two patients (6.6%) dhad functional over cysts. These cysts are smaller than 4 cm, simple and do not give pain. In NETA group at the end of the third month two patients (6.6%) had functional over cysts. All these cysts have naturally disappeared during the controls.

The other adverse effects for LNG-IUD group after three months were hirsutismus in one patient, headache in two patients, acne in one patient and pelvic pain in two patients; at the end of the sixth month hirsutism in one patient, headache in one patient, acne in one patient, pelvic pain in one patient. In NETA group at the end of the third month acne was seen in one patient, hirsutism in three patients, headache in two patients, depressive state in five patients; at the end of the sixth month acne in one patient, hirsutism in one patient, headache in one patient, depressive state in three patients. Two patients had stopped taking medicine after six months because of their depressive state. The other adverse effects disappeared after six months. 

## CONCLUSION

In addition to its high contraceptive efficacy, LNG-IUD is also used successfully for different gynecological pathologies. Since it has local effects, systemic side effects are less. Its therapeutic efficacy in heavy menstrual bleeding is considerably high. In addition, its favourable effect to improve dysmenorrhea and premenstrual symptoms increases the patients’ satisfaction. Since it has reversible effects in the women of reproductive age especially who want to keep their fertilities, it is an effective, noninvasive method in heavy menstrual bleeding treatment which provides long-term efficacy. It may also prevent needless hysterectomy and endometrial ablation in premenopausal women and so reduces postoperative mortality and morbidity. When compared with surgical treatment, its cost-effectiveness is lower.

Since oral progestagens are drugs for which patient compliance and treatment continuation rate is low during long-term treatment, it is not a preferred method of treatment.The most important limitation for LNG-IUD use is high cost and limited clinical experience.

LNG-IUD is a good alternative to oral progestagens for treatment of heavy menstrual bleeding observed in the cases with uterine myoma due to its ease of use and good patient tolerance. 

## Authors Contribution:

AKT & IT conceived, designed and did statistical analysis, editing of manuscript, did data collection and manuscript writing

NS did review and final approval of manuscript, takes the responsibility and is accountable for all aspects of the work in ensuring that questions related to the accuracy or integrity of any part of the work are appropriately investigated and resolved.
